# Therapeutic effect of whole brain radiotherapy on advanced NSCLC between EGFR TKI-naïve and TKI-resistant

**DOI:** 10.1186/s13014-019-1454-2

**Published:** 2019-12-31

**Authors:** Lihao Zhao, Xiaona Cai, Didi Chen, Xuxue Ye, Mengdan Gao, Lihuai Lu, Huafang Su, Meng Su, Meng Hou, Congying Xie

**Affiliations:** 1 Department of Radiation and Medical Oncology, the First Affiliated Hospital of Wenzhou Medical University, No.2 Fuxue Lane, Wenzhou, 325000 Zhejiang China; 2 Department of Ultrasonography, the First Affiliated Hospital of Wenzhou Medical University, Wenzhou, 325000 Zhejiang China

**Keywords:** Non-small-cell lung cancer, EGFR-TKI resistance, EGFR TKI-naïve, The treatment sequence, Whole brain radiotherapy

## Abstract

**Background:**

The development of epidermal growth factor receptor-tyrosine kinase inhibitors (EGFR-TKIs) has dramatically improved the prognosis of patients with EGFR-mutant non-small-cell lung cancer (NSCLC). The purpose of this study is to investigate the clinical outcome with or without EGFR-TKI resistance before WBRT and the sequence between EGFT-TKIs and whole brain radiotherapy (WBRT) of EGFR-mutant NSCLC patients who developed multiple brain metastases (BMs).

**Patients and methods:**

Three hundred forty-four EGFR-mutant NSCLC patients with multiple BMs were reviewed. Enrolled patients were divided into TKI-naïve group and TKI-resistant group. The intracranial progression-free survival (PFS) and overall survival (OS) were analyzed via the Kaplan-Meier method.

**Results:**

For patients with multiple BMs treated by WBRT, the median intracranial PFS and OS were longer in the TKI-naïve group than those in the TKI-resistant group, but there were no statistically significant between two groups (Intracranial PFS: 7.7 vs. 5.4 months, *p* = 0.052; OS: 11.2 vs. 9.2 months, *p* = 0.106). For patients with Lung-molGPA 0–2, no significant differences in median intracranial PFS (6.2 vs. 5.2 months, *p* = 0.123) and OS (7.8 vs. 6.7 months, *p* = 0.514) between TKI-naïve and TKI-resistant groups. For patients with Lung-molGPA 2.5–4, intracranial PFS: 12.8 vs. 10.1 months; OS: 23.3 vs. 15.3 months.

**Conclusions:**

Our study found that there were no difference in intracranial PFS and OS in all patients between the two groups of TKI-naïve and TKI-resistant. But for patients in subgroup of Lung-molGPA 2.5–4, there were a better intracranial PFS and OS in TKI-naïve group.

## Background

Lung cancer is the leading cause of cancer-associated mortality in China and worldwide, and non-small-cell lung cancer (NSCLC) represents about 80–85% of all lung cancers [1, 2]. Central nervous system (CNS) metastasis is a prevalent and serious complication of NSCLC, with negative effects on quality of life and overall survival [3]. More than 10% of NSCLC patients presented with brain metastasis (BM) at the first primary diagnosis time [4, 5] and approximately 30–40% of patients with NSCLC develop brain metastases during the course of their disease [6].

Whole brain radiotherapy (WBRT) is one of therapeutich local approach for multiple BMs [7]. However, the prognosis of patients with multiple BMs remains poor after WBRT with a median overall survival (OS) of 3–5 months, mainly because the brain metastasis can not be effectively controlled. Several chemotherapy drugs in combination with WBRT failed to improve the survival because of the impenetrability of brain blood barrier (BBB) [8]. Compared with chemotherapy, many targeted agents have been developed to improve the typically dismal outcome associated with NSCLC, especially small-molecule epidermal growth factor receptor (EGFR) tyrosine kinase inhibitors (TKI) for its capability of crossing the BBB. In particular, the third generation of EGFR-TKI (Tagrisso) has been shown to have a good ability to control BM for patient with EGFR-mutant NSCLC [9, 10]. In the era of targeted-therapy, the development of EGFR-TKIs has dramatically improved the prognosis of patients with EGFR-mutant NSCLC. These agents improve response rates (RR), prolonged progression-free survival (PFS), and overall survival (OS). However, the majority of patients who initially respond to TKI therapy will finally develop resistance to TKIs, limiting patient benefit and posing a challenge to oncologists [11]. Furthermore, the initial failure site for acquired resistance to TKIs is usually in the CNS [12]. The management of brain metastases persists as an important issue. Radiotherapy is considered as an effective therapy for brain metastases, which has yielded response rates of 50 to 75% for intracranial lesions, especially for those developed acquired resistance to TKIs [13, 14].

At present, whether acquired resistance to EGFR-TKI affects the efficacy of RT in NSCLC patients remains controversial. Currently, some scholars believe that targeted therapy synchronous WBRT may reduce the OS of patients with NSCLC with multiple BMs accompanied by EGFR mutations. Therefore, sequential therapy may be a better treatment option. However, there is still a controversy over whether to receive TKI therapy after radiotherapy or TKI therapy before radiotherapy. To clarify the influence of acquired EGFR-TKI resistance on the efficacy of radiotherapy in the treatment of NSCLC patients with multiple BMs, and how to choose the treatment sequence between the two approaches, we assessed the clinical outcomes of WBRT in the treatment of EGFR-mutant brain metastatic NSCLC patients with or without EGFR-TKI resistance.

## Patients and methods

### Patients

Clinical data of NSCLC patients with multiple BMs harboring EGFR mutation at the authors’ hospital from January 2008 to March 2018 were retrospectively reviewed. The eligibility criteria for this study was set as following: patients were diagnosed with NSCLC and confirmed multiple BMs by magnetic resonance imaging (MRI); patients had at least four BMs and headaches, accompanied by nausea, vomiting or visual disorder, or dizziness, tinnitus, deafness and other symptoms; patients had at least four measurable BMs according to the Response Evaluation Criteria in Solid Tumors (RECIST) 1.1; the EGFR mutations include exons 18 to 21, deletion on exon 19 and point mutation on exon 21; patients had no serious dysfunction of major organs (e.g. heart failure or uremia); patients had adequate function of hematologic (absolute neutrophil ≥1.5*10^9^/L or platelet count ≥100*10^9^/L); patients treated by WBRT for a prescription of 3 Gy *10 fractions;

The exclusion criteria was set as following: patients had mixed small cell carcinoma or small cell carcinoma histologically; Patients had less than four measurable BMs lesion according to the RECIST 1.1; patients were lost to follow-up or died within 1 month after starting the treatment; patients received prior radiotherapy or targeted drugs for BM or temozolomide (TMZ); patients received concurrent radiochemotherapy; patients received radiosurgery (SRS) or surgery for BM. This study was approved by the Institutional Review Board and performed at the 1st Affiliated Hospital of Wenzhou Medical University.

### The parameters of the Lung-mol GPA

The prognostic groupings of the denocarcinoma Lung-molGPA was a predictor of NSCLC patient with multiple BMs. Including the following five factors: Age, KPS, Extracranial Metastases, Number of BM, Gene status. The parameters of the Lung-molGPA were detailed in Table 1. Scores can be divided into 0–4, and lower scores that means the prognosis would be worse.

**Table 1:** Lung-molGPA for NSCLC with BM Scoring Chart

Prognostic Factor	Lung-molGPA		
	0	0.5	1
Age, y	≥70	< 70	
KPS	< 70	70–80	90–100
Extracranial Metastases	Present		Absent
Number of BM	> 4	1–4	NA
Gene status	EGFR neg/unk and ALK neg/unk	NA	EGFR pos or ALK pos

*Abbreviations*: *NSCLC* Non-small-cell lung cancer, *BM* brain metastasis, *Lung-molGPA* Lung Cancer Molecular Markers Graded Prognostic Assessment, *KPS* Karnofsky Performance Status, *NA* not applicable, *neg/unk* negative or unknown, *pos* positive

### Study design

Eligible patients were divided into two groups: TKI-naïve group and TKI-resistant group. In TKI-naïve group, patients were accepted initial TKI treatment and no TKI-resistant were found before WBRT. In TKI-resistant group, patients harboring EGFR mutation, and experienced disease progression after initial benefit from TKIs or with newly-diagnosed brain metastases during TKIs treatment and/or salvage chemotherapy. Patients with different Lung Cancer Using Molecular Markers Graded Prognostic Assessment (Lung-molGPA) were further divided into two subgroups of Lung-molGPA 0–2 (with Lung-molGPA value from 0 to 2) and Lung-molGPA 2.5–4 (with Lung-molGPA value from 2.5–4).

WBRT was planned by two lateral parallel-opposite conformal beams with a prescription of 30 Gy at 10 fractions for a 6-MV photon beam on an Elekta Synergy® linac (Elekta Ltd., Crawley, UK). To minimize the side effects of WBRT, we used side-to-side radiation and intensity modulated radiation therapy (IMRT). Beyond that, with the development of radiotherapy technology, some patients used new radiotherapy technology which penetrating field to protect the hippocampal gyrus. (It can form a lower dose distribution in the hippocampus to achieve the purpose of protecting the hippocampus, and can effectively protect the hippocampus without reducing the dose of intracranial lesions.)

All patients were evaluated weekly during WBRT. They were followed up every 3 months for 1–3 years after the end of radiotherapy and every 6 months thereafter until death or the deadline of our study. Evaluation included a complete history review, neurologic examination, blood counts, and biochemistry profile. During follow-up evaluation including physical examination, neurologic examination, a complete blood count measurement, liver function test, and chest computed tomography (CT) scan was done monthly. Brain CT with and without contrast, abdominal CT, or bone scan, as well as MRI if necessary, were performed when there were relevant symptoms in patients.

### Statistical analyses

Pearson chi-square or Fisher’s exact tests (when there were fewer than 5 expected counts in the contingency table) were used to compare the baseline characteristics of parents between TKI-naïve and TKI-resistant groups. Tumor response was assessed according to the RECIST 1.1. OS was defined as the interval from the date of initial diagnosis of brain metastasis to the date of death resulted. Intracranial PFS was defined as interval between the WBRT initiation and the date of confirming CNS progression or death from CNS progression, if death occurred within 60 days of the last CNS assessment date. If the patient’s complete follow up data was impossible to obtain or the disease did not progress, patients’ status was assumed as the last known survival and/or contact date. Adverse events were graded according to the National Cancer Institute Common Terminology Criteria for Adverse Events (NCI-CTCAE) v4.0.

Intracranial PFS and OS were analyzed using the Kaplan-Meier method. Differences between two groups were compared by the log-rank test. The Cox proportional hazards model was used for univariate and multivariate analyses to identify the independent prognostic factors for PFS and OS. Statistical analyses were carried out with SPSS 22.0 software. Tests were two sided. *P*-value < 0.05 was considered statistically significant, and robust estimates of the standard error were used in all regression analyses.

## Results

### EGFR mutations analyses

Patients’ specimens for genetic test were obtained from primary tumor or metastatic sites through either diagnostic or surgical procedures. All samples were paraffin-embedded material. Tumor cells were isolated using micro dissection to ensure their presence in the specimens for DNA sequencing. EGFR gene was amplified by polymerase chain reaction and somatic mutations was detected using direct sequencing. The TKI drugs used by the patients were the first and second generation TKI drugs. Osimertinib was not included in this study because most patients were enrolled early.

### Patient characteristics

Among total 478 NSCLC patients with multiple BMs harboring EGFR mutations in hospital during January 2008 to March 2018, 344 patients were enrolled in this study. Thirty-one patients who were lost to follow-up, 14 patients who received EGFR TKIs and 18 patients who received TMZ before WBRT, and 42 patients without WBRT were excluded. In addition, 21 patients who received second time EGFR-TKIs or pemetrexed, 8 patients had operations or SRS to treat brain metastases were also excluded. Of the 344 enrolled patients, the number of patients in Lung-molGPA 0–2 group and Lung-molGPA 2.5–4 group was 199 (57.8%) and 145 (42.2%), respectively. The percentage of patients with Lung-molGPA 0–2 group and Lung-molGPA 2.5–4 group for TKI-naïve and TKI-resistance were 71.4, 28.6 and 44.8%, 55.2%, respectively.

Patients in Lung-molGPA 0–2 group and Lung-molGPA 2.5–4 group between the TKI-naïve and TKI-resistant groups were well balanced with regard to gender, smoking, age, histology type, KPS, number of BM, extracranial metastases, primary disease control (Table 2).

**Table 2:** Clinical characteristics of non-small cell lung cancer patients with brain metastases harboring EGFR mutations

Characteristics	All patients			Patients with Lung-molGPA 0–2			Patients with Lung-molGPA 2.5–4		
	TKI-naïve group (%)	TKI-resistant group (%)	*p*	TKI-naïve group (%)	TKI-resistant group (%)	*p*	TKI-naïve group (%)	TKI-resistant group (%)	*p*
All patients	207 (100)	137 (100)		142 (100)	57 (100)		65 (100)	80 (100)	
Gender									
Female	72 (34.8)	53 (38.7)		45 (31.7)	21 (36.8)		27 (41.5)	32 (40.0)	
Male	135 (65.2)	84 (61.3)	0.461	97 (68.3)	36 (63.2)	0.485	38 (58.5)	48 (60.0)	0.851
Smoking									
Never	97 (46.9)	68 (49.6)		60 (42.3)	26 (45.6)		37 (56.9)	42 (52.5)	
Current/former	110 (53.1)	69 (50.4)	0.614	82 (57.7)	31 (54.4)	0.665	28 (43.1)	38 (47.5)	0.595
Age									
< 70	162 (78.3)	112 (81.8)		104 (73.2)	43 (75.4)		58 (89.2)	69 (86.3)	
≥ 70	45 (21.7)	25 (18.2)	0.431	38 (26.8)	14 (24.6)	0.747	7 (10.8)	11 (13.7)	0.588
Histology									
Adenocarcinoma	116 (56.0)	90 (65.7)		78 (54.9)	34 (59.6)		38 (58.5)	56 (70.0)	
Non-adenocarcinoma	91 (44.0)	47 (34.3)	0.074	64 (45.1)	23 (40.4)	0.544	27 (41.5)	24 (30.0)	0.148
KPS									
< 70	84 (40.6)	57 (41.6)		70 (49.3)	36 (63.2)		14 (21.5)	21 (26.3)	
70–100	123 (59.4)	80 (58.4)	0.850	72 (50.7)	21 (36.8)	0.076	51 (78.5)	59 (73.7)	0.510
Number of BM									
1–4	124 (59.9)	84 (61.3)		80 (56.3)	24 (42.1)		44 (67.7)	60 (75.0)	
> 4	83 (40.1)	53 (38.7)	0.793	62 (43.7)	33 (57.9)	0.069	21 (32.3)	20 (25.0)	0.331
Extracranial metastases									
Absent	81 (39.1)	47 (34.3)		23 (16.2)	8 (14.0)		58 (89.2)	39 (48.8)	
Present	126 (60.9)	90 (65.7)	0.365	119 (83.8)	49 (86.0)	0.704	7 (10.8)	41 (51.2)	0.352
Primary disease control									
No	94 (45.4)	63 (46.0)		72 (50.7)	32 (56.1)		22 (33.8)	31 (38.8)	
Yes	113 (54.6)	74 (54.0)	0.917	70 (49.3)	25 (43.9)	0.488	43 (66.2)	49 (61.2)	0.542

*Abbreviations*: *NSCLC* Non-small-cell lung cancer, *BM* brain metastasis, *Lung-molGPA* Lung Cancer Molecular Markers Graded Prognostic Assessment, *TKI* tyrosine kinase inhibitors, *EGFR* epidermal growth factor receptor, *KPS* Karnofsky Performance Status

### Outcomes stratified by groups

**Fig. 1:**
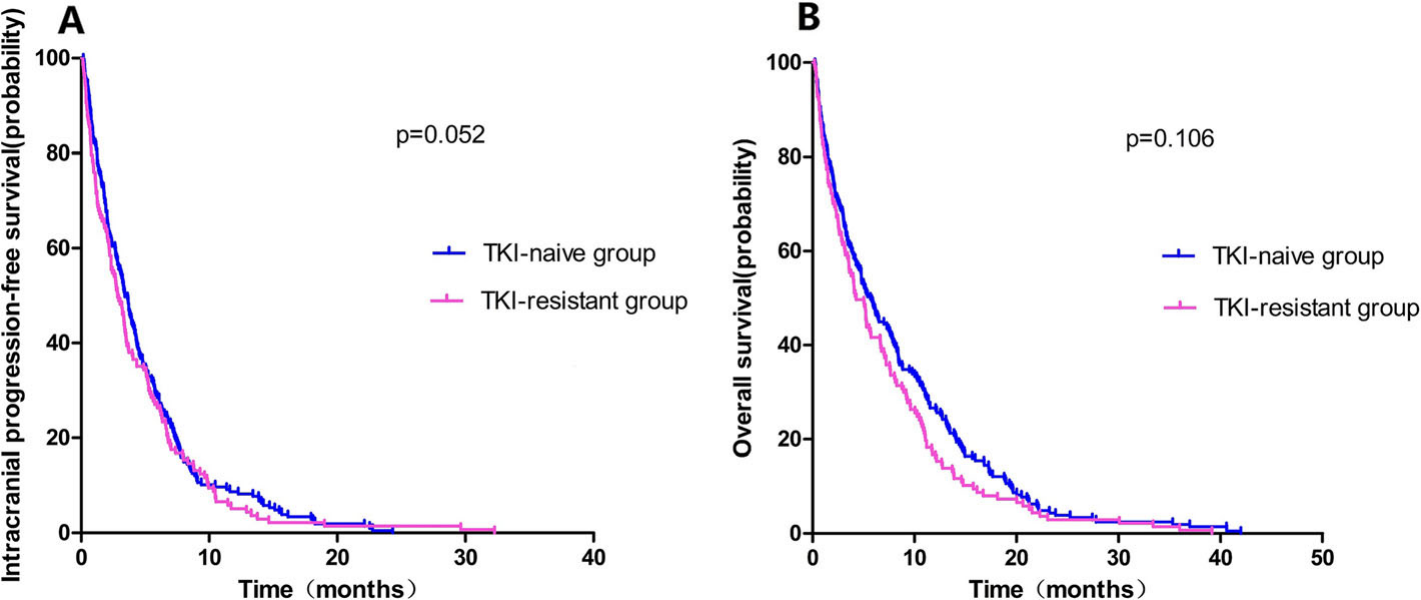
Intracranial Progression-free Survival (**a**) and Overall Survival (**b**) of all NSCLC developed multiple BMs patients

**Fig. 2:**
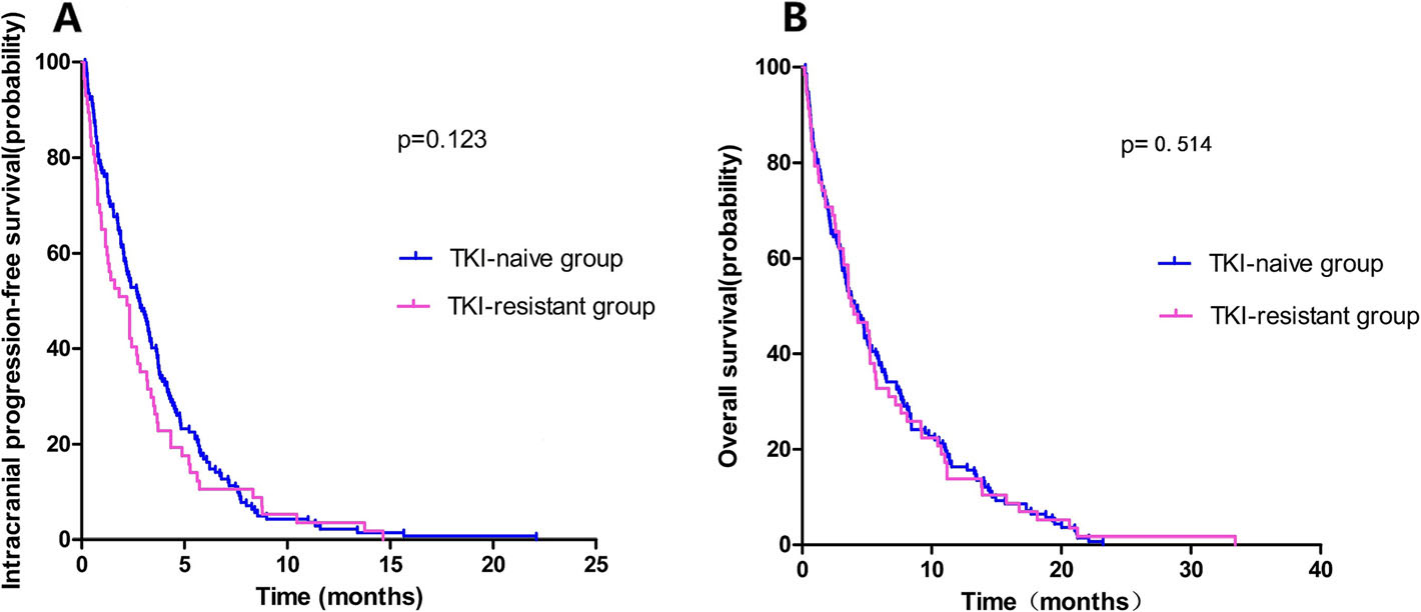
Intracranial Progression-free Survival (**a**) and Overall Survival (**b**) of NSCLC developed multiple BMs patients with Lung-molGPA 0–2

**Fig. 3:**
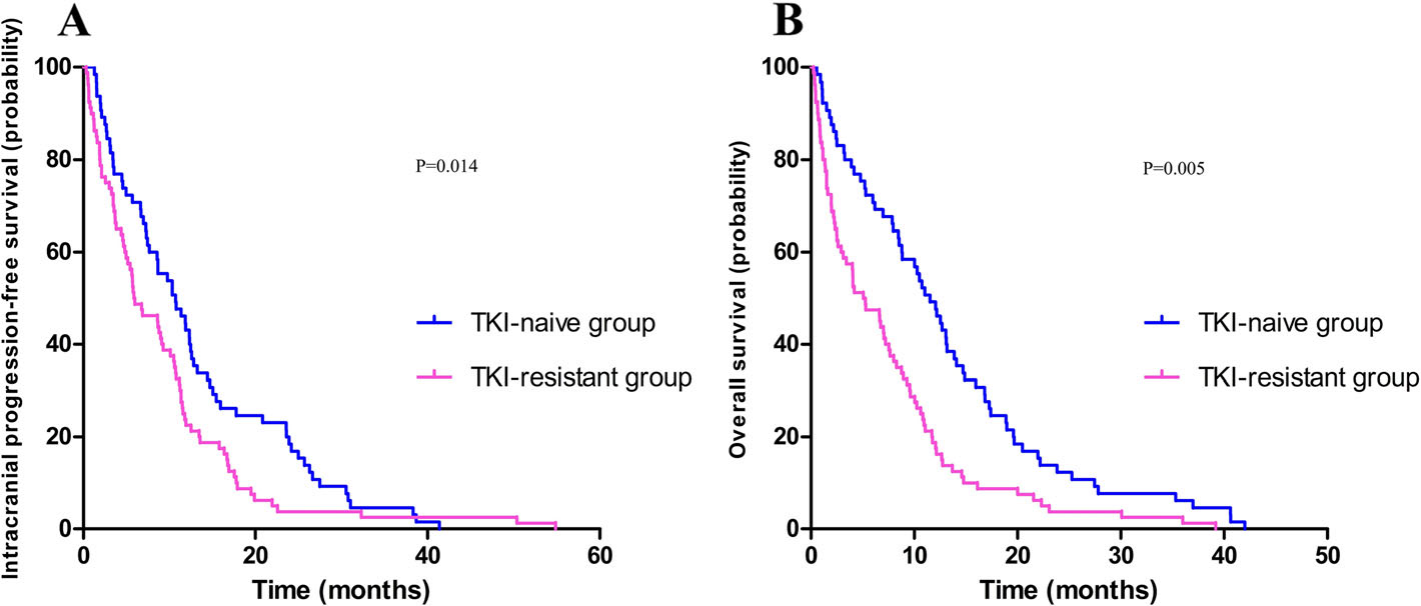
Intracranial Progression-free Survival (**a**) and Overall Survival (**b**) of NSCLC developed multiple BMs patients with Lung-molGPA 2.5–4

The median intracranial PFS and OS for TKI-naïve group of all enrolled NSCLC patients were 7.7 months (95% Cl, 6.6–8.7 months) and 11.2 months (95% Cl, 7.7–14.6 months). As for TKI-resistant group, the median intracranial PFS and OS were 5.4 months (95% Cl, 4.0–6.7 months) and 9.2 months (95% Cl, 6.1–12.3 months), respectively. The PFS and OS of all enrolled patients are presented in Fig. 1. The TKI-naïve group had a longer median intracranial PFS (7.7 vs. 5.4 months, *p* = 0.052) and OS (11.2 vs. 9.2 months, *p* = 0.106) compared with the TKI-resistant group, but there were no statistically significant between two groups. Of the 199 patients in Lung-molGPA 0–2 group, 142 (71.4%) and 57 (28.6%) patients were in the TKI-naïve group and TKI-resistant arms. The intracranial PFS and OS of Lung-molGPA 0–2 group patients are presented in Fig. 2. The TKI-naïve group had no significant differences between the two groups in intracranial PFS (6.2 vs. 5.2 months, *p* = 0.123) and OS (7.8 vs. 6.7 months, *p* = 0.514). Of the 145 patients in Lung-molGPA 2.5–4 group, 65 (44.8%) and 80 (55.2%) patients were in the TKI-naïve group and TKI-resistant group. As shown in Fig. 3, the TKI-naïve arm had a significant longer median intracranial PFS (12.8 vs. 10.1 months, *p* = 0.014) and OS (23.3 vs. 15.3 months, *p* = 0.005) than the TKI-resistant group.

The outcomes above showed that patients with Lung-molGPA 2.5–4 and without EGFR-TKIs treatment before WBRT had the best intracranial PFS and OS. (12.8 months, 95% CI 10.3–15.4, and 23.3 months, 18.5–28.1, respectively).

### Multivariate analysis and toxicities

Multivariate analysis on intracranial PFS and OS for all NSCLC patients and for patients with Lung-molGPA 0–2 and Lung-molGPA 2.5–4 groups were shown in Table 3. For patients with Lung-molGPA 2.5–4 groups, age (*p* = 0.01), extracranial metastases (*p* = 0.04) were associated with OS according to multivariate analysis.

**Table 3:** Multivariate analysis of factors affecting intracranial PFS and OS in the patients

Factors	Intracranial PFS			OS		
	HR	95%CI	p	HR	95%CI	p
All patients						
Gender	1.10	0.50–2.41	0.82	0.67	0.33–1.36	0.27
Smoking	1.12	0.53–2.38	0.77	0.84	0.44–1.63	0.61
Age	0.99	0.65–1.52	0.97	1.40	0.33–1.36	0.11
Histology	0.82	0.54–1.23	0.33	1.40	0.93–2.13	0.11
KPS	0.90	0.58–1.40	0.63	0.97	0.65–1.46	0.88
Number of BM	0.89	0.56–1.41	0.61	0.88	0.57–1.35	0.55
Extracranial metastases	0.96	0.53–1.74	0.89	1.15	0.66–2.01	0.63
Primary disease control	0.10	0.93–2.16	0.10	0.92	0.61–1.38	0.68
Patients with Lung-molGPA 0–2						
Gender	1.04	0.52–2.09	0.91	1.00	0.44–2.27	1.00
Smoking	0.82	0.40–1.68	0.59	0.98	0.43–2.20	0.95
Age	0.85	0.52–2.09	0.57	1.32	0.75–2.30	0.33
Histology	0.91	0.52–1.56	0.72	0.65	0.37–1.14	0.13
KPS	0.61	0.32–1.15	0.13	1.38	0.76–2.50	0.30
Number of BM	0.79	0.47–1.33	0.38	1.26	0.74–2.15	0.40
Extracranial metastases	0.64	0.37–1.11	0.11	1.06	0.58–1.93	0.86
Primary disease control	1.39	0.83–2.32	0.21	0.88	0.49–1.59	0.67
Patients with Lung-molGPA 2.5–4						
Gender	1.11	0.66–1.85	0.70	0.81	0.50–1.31	0.39
Smoking	1.07	0.65–1.75	0.80	0.89	0.56–1.42	0.63
Age	1.07	0.77–1.48	0.70	1.49	1.09–2.05	0.01
Histology	0.79	0.58–1.09	0.15	1.12	0.81–1.54	0.51
KPS	1.02	0.74–1.39	0.93	1.06	0.78–1.43	0.73
Number of BM	0.97	0.71–1.34	0.86	0.98	0.72–1.34	0.91
Extracranial metastases	1.19	0.87–1.64	0.28	1.40	1.01–1.93	0.04
Primary disease control	1.37	0.10–1.89	0.05	1.04	0.76–1.42	0.81

*Abbreviations*: *PFS* progression-free survival, *OS* overall survival, *HR* hazard ratio, *CI* confidence interval, *KPS* Karnofsky Performance Status, *BM* brain metastasis, *Lung-molGPA*, Lung Cancer Molecular Markers Graded Prognostic Assessment

Toxicities were reported in all NSCLC patients and for patients with Lung-molGPA 0–2 and Lung-molGPA 2.5–4 groups were the most frequent observed hematologic side effects, as showed in Table 4. The common grade III/IV toxicity were nausea (*N* = 142, 68.6% and *N* = 95, 69.3%) and vomiting(*N* = 134, 64.7% and *N* = 83, 60.6%). Most patients tolerated well with the side effects of EGFR-TKIs and WBRT after symptomatic treatments. Overall, all toxicities were generally brief, reversible, and manageable. They were well tolerated after symptomatic treatments.

**Table 4:** Toxicity profile for all patients

Side effects	TKI-naïve (%) (*N* = 207)		TKI-resistant (*N* = 137)	
	All grades, N. (%)	Grade III/IV, N. (%)	All grades, N. (%)	Grade III/IV, N. (%)
Fatigue	117 (56.5)	19 (9.1)	81 (59.1)	14 (10.2)
Anorexia	84 (40.5)	18 (8.7)	56 (40.9)	10 (7.3)
Diarrhea	23 (11.1)	4 (0.2)	12 (8.8)	1 (0.7)
Nausea	142 (68.6)	38 (18.4)	95 (69.3)	23 (16.8)
Vomiting	134 (64.7)	15 (7.2)	83 (60.6)	15 (10.9)
Headache	105 (50.7)	14 (6.8)	51 (37.2)	12 (8.8)
Anemia	113 (54.6)	5 (2.4)	75 (54.1)	3 (2.2)
Neutropenia	95 (45.9)	17 (8.2)	69 (50.4)	11 (8.0)
Thrombocytopenia	88 (42.5)	6 (2.9)	62 (45.3)	2 (1.5)

*Abbreviations*: *TKI* tyrosine kinase inhibitors

## Discussion

The efficacy of WBRT in the treatment of advanced NSCLC patients with multiple BMs was investigated in a total of 344 patients with or without acquired resistance to TKIs. The median OS in this study (11.2 and 9.2 months for TKI-naïve and TKI-resistant group, respectively) for patients were longer than reported median OS of 3–5 months [7, 15, 16]. The improved OS could be due to that all patients enrolled in this study were EGFR-mutant. Das AK et al. observed that NSCLC cells with EGFR-mutant had a better radiosensitivity compared with those with EGFR wild-type in vitro [17]. Similarly, Lee HL et al. reported that patients with mutant EGFR had higher response rates to brain radiotherapy than those with wild-type EGFR (80% vs. 46%, *p* = 0.037), and EGFR mutation status was the only predictor for treatment response (*p* = 0.032) [18]. In this study, subgroup analysis showed a better intracranial PFS and OS in Lung-molGPA 2.5–4 group compared with Lung-molGPA 0–2. Lung-molGPA score was also a significant prognostic predictor. Paul W. et al. indicated that the updated Lung-molGPA was associated with improved prognosis by incorporating the effect of EGFR and anaplastic lymphoma kinase (ALK) gene alterations on survival in NSCLC patients with BMs, comparison with Oncology Group Recursive Partitioning Analysis (RTOG RPA) and the original Diagnosis-Specific Graded Prognostic Assessment (DS-GPA) [19].

In the era of targeted-therapy, the development of EGFR-TKIs has dramatically improved the prognosis of NSCLC patients with EGFR mutantation. The ability of EGFR inhibitors to enhance radiation antitumor activity has been reported as well [20–22]. In addition, studies have shown that EGFR-positive NSCLC patients had a higher radiosensitivity. Das et al. have shown that EGFR-mutant NSCLC cells are more sensitive to RT than EGFR wild-type NSCLC cells [17]. However, some studies had the opposite opinion and that suggest patients with EGFR mutations are resistant to RT. Kosaka T et al. observed that MET amplification, overexpression of hepatocyte growth factor and secondary threonine-to-methionine mutation at codon 790 in exon 20 of the EGFR gene may be involved in radiation resistance [23]. Previous studies suggested that the first-line treatment of patients with advanced, recurrent, or metastatic NSCLC harboring an EGFR mutation is an EGFR-TKI [24]. A recent study, An N et al. point that first-line TKIs plus concurrent cranial radiotherapy is a promising therapeutic strategy that led to remarkable intracranial PFS improvement and survival benefits for EGFR-mutant NSCLC with BM [25]. However, lots of patients who initially respond to TKI therapy will finally develop resistance to TKIs [13]. Current guidelines recommend patients to continued use TKI after TKI-resistant for NSCLC patient with brain metastasis [26]. However, many studies have shown that acquired resistance to TKIs seems to be associated with worse efficacy of RT for brain metastases from EGFR-mutant NSCLC [26–29]. Magnuson WJ et al. found that acquired EGFR-TKI resistance may result in inferior intracranial PFS and OS compared with those using upfront radiotherapy for patients with EGFR-mutant NSCLC [29]. At present, there was no definite conclusion on whether TKI resistance could affect the efficacy of RT [11]. The purpose of this study is to investigate the clinical outcome with or without EGFR-TKI resistance before WBRT of EGFR-mutant NSCLC patients who developed BMs. This study demonstrated that there were no difference in intracranial PFS and OS in all patients between the two groups of TKI-naïve and TKI-resistant.

Previous studies suggested that the first-line treatment of patients with advanced, recurrent, or metastatic NSCLC harboring an EGFR mutation is an EGFR-TKI [24]. Whole brain radiotherapy (WBRT) is one of the effective control methods for multiple BMs [7]. Chen CH et al. Chen CH er al. found that the negative survival impact from the omission of WBRT in patients with EGFR-mutant NSCLC [30]. However, the sequencing between RT and TKIs remains an issue. Currently, it has been suggested that targeted therapy synchronous WBRT may reduce OS in patients with NSCLC who are diagnosed with multiple BMs accompanied by EGFR mutations. Therefore, sequential therapy may be a better treatment option. However, there is still a controversy over whether TKI therapy should be followed by radiotherapy first or TKI therapy first [27]. Our study demonstrated that patients with EGFR-TKIs resistance had a worse intracranial PFS and OS in Lung-molGPA 2.5–4 group (*p* = 0.005), compared with those without EGFR-TKIs resistance, but there were no significant differences in Lung-molGPA 0–2 group. Magnuson WJ et al. reached a similar conclusion. Patients were grouped according to the GPA, they found that patients with acquired EGFR TKI-resistance have a poor OS (78.9 vs. 19.5 months, *p* < 0.01) than TKI-naïve patients in GPA 2.5–4 group [29]. Li C et al. came to the same opinion. They think that the use of upfront WBRT for EGFR-mutated lung adenocarcinoma patients with multiple BM can improve ORR and OS [31]. Our data showed that the use of RT to TKI-naïve NSCLC result in better intracranial PFS and OS for patients with Lung-molGPA 2.5–4, TKI-naïve may be a beneficial prognostic factor. Based on the above researchs, we propose for patients with Lung-molGPA 2.5–4 that upfront WBRT followed by EGFR-TKI therapy would be a better treatment strategy in the treatment for EGFR-mutant NSCLC with symptomatic multiple BMs.

Osimertinib was not included in the study, mainly because it was enrolled earlier since 2008. At present, some studies have shown that osimertinib has radiotherapy sensitization effect. Wang N et al. [32] observed that osimertinib has therapeutic potential as a radiation-sensitizer in lung cancer cells harboring the EGFR T790 M mutation. This is also a new direction of our research.

There are some limitations in this study: first, this study was designed retrospectively from a single-institution, which may experienced inherent bias, second, the number of patients enrolled in this study may be not sufficient. Factors that may impact the outcomes could not be fully evaluated. The number of patients enrolled in this study may not be sufficient enough and the follow-up period may be not long enough. External validation using other large database to further evaluate the prognostic effect of radiotherapy in the treatment of TKI-resistant NSCLC patients with multiple BMs would be of great value in clinical practice.

EGFR-TKI was the first-line treatment of patients with advanced, recurrent, or metastatic NSCLC harboring an EGFR mutation. At present, there was no definite conclusion on whether TKI resistance could affect the efficacy of RT.

## Conclusions

Our study found that there were no difference in intracranial PFS and OS in all patients between the two groups of TKI-naïve and TKI-resistant. But for patients in subgroup of Lung-molGPA 2.5–4, there were a better intracranial PFS and OS in TKI-naïve group. However, the treatment sequence of the two treatment methods is still controversial, more large randomized clinical trials are needed for further verification.

## Data Availability

The datasets used and analysed during the current study are available from the corresponding author on reasonable request.

## Abbreviations

ALK: Anaplasticlymphoma kinase
BBB: Brain blood barrier
BM: Brain metastasis
CNS: Central nervous system
CT: Computed tomography
DS-GPA: Diagnosis-Specific Graded Prognostic Assessment
EGFR: Epidermal growth factor receptor
IMRT: We used intensity modulated radiation therapy
KPS: Karnofsky Performance Status
Lung-molGPA: Lung Cancer Molecular Markers Graded Prognostic Assessment
MRI: Magnetic resonance imaging
NA: Not applicable
NCI-CTCAE: National Cancer Institute Common Terminology Criteria for Adverse Events
neg/unk: Negative or unknown
NSCLC: Non-small-cell lung cancer
OS: Overall survival
PFS: Progression-free survival
pos: Positive
RECIST: Response Evaluation Criteria in Solid Tumors
RTOG RPA: Oncology Group Recursive Partitioning Analysis
SRS: Radiosurgery
TKI: Tyrosine kinase inhibitors
TMZ: Temozolomide
WBRT: Whole-brain radiotherapy
